# Echocardiographic screening for heart failure and optimization of the care pathway for individuals with pacemakers: a randomized controlled trial

**DOI:** 10.1038/s41591-024-03265-3

**Published:** 2024-09-19

**Authors:** Maria F. Paton, John Gierula, Haqeel A. Jamil, Sam Straw, Judith E. Lowry, Rowena Byrom, Thomas A. Slater, Alasdair M. Fellows, Richard G. Gillott, Hemant Chumun, Paul Smith, Richard M. Cubbon, Deborah D. Stocken, Mark T. Kearney, Klaus K. Witte

**Affiliations:** 1 Leeds Institute of Cardiovascular and Metabolic Medicine, University of Leeds, Leeds, UK; 2 Leeds Teaching Hospitals NHS Trust, Department of Cardiology, Leeds, UK; 3 Leeds Institute of Clinical Trials Research, University of Leeds, Leeds, UK; 4 Department of Cardiology, Bradford Teaching Hospitals NHS Trust, Bradford, UK

**Keywords:** Outcomes research, Heart failure, Cardiac device therapy

## Abstract

Individuals with pacemakers are at increased risk of left ventricular systolic dysfunction (LVSD). Whether screening for and optimizing the medical management of LVSD in these individuals can improve clinical outcomes is unknown. In the present study, in a multicenter controlled trial (OPT-PACE), we randomized 1,201 patients (717 men) with a pacemaker to echocardiography screening or usual care. In the screening arm, LVSD was detected in 201 of 600 (34%) patients, who then received management in either primary care or a specialist heart failure (HF) and devices clinic. The primary outcome of the trial was the difference in a composite of time to first HF hospitalization or death. Over 31 months (interquartile range = 30–40 months), the primary outcome occurred in 106 of 600 (18%) patients receiving echocardiography screening, which was not significantly different compared with the occurrence of the primary outcome in 115 of 601 (19%) patients receiving the usual care (hazard ratio = 0.89; 95% confidence interval = 0.69, 1.17). In a prespecified, nonrandomized, exploratory analysis, patients with LVSD managed by the specialist clinic experienced the primary outcome event less frequently than those managed in primary care. The results of this trial indicate that echocardiography screening commonly identifies LVSD in individuals with pacemakers but alone does not alter outcomes. ClinicalTrials.gov registration: NCT01819662.

## Abstract (web summary)

For individuals with pacemakers, a care pathway that includes echocardiographic screening to detect signs of heart failure did not improve cardiac outcomes, but patients flagged as having impaired heart function who were managed by a specialized heart failure clinic benefited, as compared to those managed by primary care physicians.

## Main

Pacemaker implantation for bradycardia improves quality of life and survival^1,2^. Over a million devices are implanted globally each year^3–5^. Long-term right ventricular (RV) pacing is associated with an adverse effect on left ventricular (LV) function and the development of HF, especially in the presence of other cardiovascular morbidities^6,7^, which augurs a worse prognosis^8–10^. Routine pacemaker follow-up offers the opportunity to screen for pacemaker-associated HF. However, people with pacemakers were either actively excluded from randomized controlled trials assessing medical therapies for HF with reduced ejection fraction (HFrEF) or subgroup analyses of those with pacemakers were not undertaken. Therefore, as a result of the lack of evidence, no medical treatment strategy has been recommended for this group in current HF or pacing guidelines^11^, such that the effects of a pathway, which includes screening followed by patient-centered education and optimized medical management for people found to have impaired LV function, are unknown.

OPT-PACE (OPTimizing PACEmaker therapy) was designed to determine the effect on clinical outcomes of screening for impaired LV function in people with pacemakers implanted for bradycardia.

## Results

**Extended Data Fig. 1:**
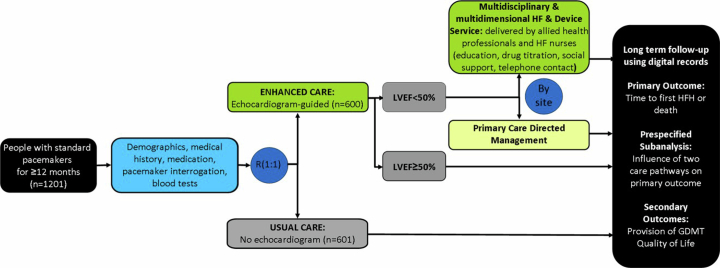
Screening for heart failure and optimizing pathways of care in people with pacemakers: The OPT-PACE randomized controlled trial. Graphical abstract describing study design, study flow, data collection time points, outcome sources and endpoints.

OPT-PACE was a randomized controlled trial that recruited patients, not previously known to have LV dysfunction, implanted with a standard pacemaker for >12 months, who were attending routine pacemaker follow-up at three hospitals in the United Kingdom. Consecutive, unselected patients agreeing to participate were randomly allocated to echocardiography screening or usual care (Extended Data Fig. 1). Depending on the site, patients found to have LV systolic dysfunction (LVSD) defined as an LV ejection fraction (LVEF) < 50% were referred to either their primary care team for management of this or a specialized, multidisciplinary, combined HF and devices service. All patients were followed up for a minimum of 12 months to establish time to a combined primary endpoint of death or HF hospitalization (HFH). Secondary endpoints included attainment of guideline-directed medical therapy for HF and quality of life. A prespecified (nonrandomized) exploratory endpoint compared outcomes in patients managed by their primary care service or the specialized clinic.

### Patient disposition

**Fig. 1:**
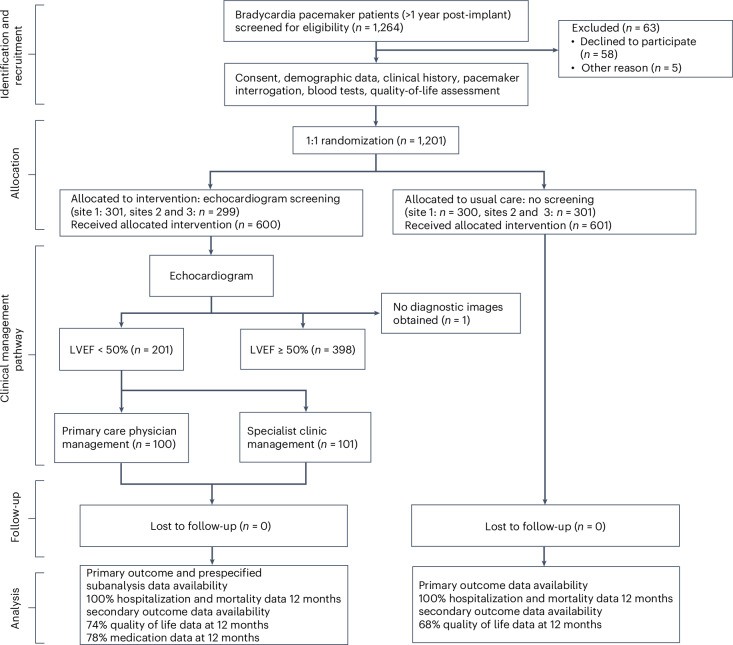
OPT-PACE CONSORT diagram. Disposition and flow of participants enrolled to OPT-PACE.

A total of 1,201 people were recruited between 1 June 2013 and 15 November 2016. Of the 1,201, 600 were randomized to echocardiography screening and 601 to the usual care (Fig. 1).

Demographic and clinical characteristics were balanced across randomized groups (Tables 1 and 2). More than half (60%) were male and the mean (s.d.) age of participants was 75 (12) years. Comorbidities included type 2 diabetes mellitus (21%), a history of myocardial infarction (18%), coronary artery bypass grafting (9%) and percutaneous coronary intervention (9%). Mean (s.d.) time since first pacemaker implantation was 7.2 (6.2) years and mean (s.d.) atrial and ventricular pacing percentages were 33% (35%) and 40% (42%), respectively.

**Table 1:** Patient demographics and characteristics at baseline

	Echocardiography screening (*n* = 600)	No echocardiography screening (*n* = 601)	Total (*n* = 1,201)
**Patient distribution by site**			
Site 1, *n* (%)	301 (50)	300 (50)	601 (50)
Site 2, *n* (%)	148 (25)	152 (25)	300 (25)
Site 3, *n* (%)	150 (25)	150 (25)	300 (25)
**Patient demographics**			
Male sex, *n* (%)	358 (60)	359 (60)	717 (60)
Age (years)	74.9 (12.2)	75.5 (11.9)	75.2 (12.0)
Height (cm)	167 (13)	166 (14)	167 (14)
Weight (kg)	78 (16)	77 (17)	78 (17)
Atrial rhythm			
Atrial fibrillation, *n* (%)	194 (32)	162 (27)	356 (30)
Paced, *n* (%)	46 (8)	62 (10)	108 (9)
Sinus rhythm, *n* (%)	359 (60)	62.79 (63)	737 (61)
**Clinical history data**			
IHD, *n* (%)	206 (34)	208 (35)	414 (35)
Diabetes mellitus			
Type 2, *n* (%)	119 (20)	128 (21)	247 (21)
Type 1, *n* (%)	3 (0.5)	3 (0.5)	6 (0.5)
CVA, *n* (%)	100 (17)	90 (15)	190 (16)
**Haemodynamic data**			
Resting heart rate (beats per min)	69 (12)	69 (12)	69 (12)
Resting systolic BP (mmHg)	138 (22)	138 (24)	138 (23)
**Pacing data**			
Pacing indication			
Atrioventricular block, *n* (%)	212 (35.6)	207 (34.3)	419 (34.9)
Sinus node disease, *n* (%)	321 (53.5)	322 (53.5)	643 (53.5)
Other, *n* (%)	67 (11.1)	76 (12.6)	143 (11.9)
Longevity of pacing (years)	7.2 (6.0)	7.2 (6.4)	7.2 (6.2)
Atrial fibrillation burden (%)	30 (45)	28 (43)	29 (44)
Atrial pacing burden (%)	32 (35)	33 (35)	32 (35)
Ventricular pacing burden (%)	41 (43)	38 (42)	40 (42)
Base rate (beats per min)	56 (8)	56 (8)	56 (7)
**Echocardiographic data**			
LVEF (%)	50 (10)		
LVEDD (mm)	47 (7)		

Continuous data are expressed as mean (s.d.) and categorical data as *n* (%) as indicated.

CVA, cerebrovascular attack; LVEDD, left ventricular end-diastolic diameter.

**Table 2:** Drug and device treatment of surviving patients by screening arm at baseline and after 12 months of follow-up

Medical therapy	Echocardiography screening (*n* = 600)		No echocardiography screening (*n* = 601)	
	Baseline (*n* = 600)	Follow-up (*n* = 468)	Baseline (*n* = 601)	Follow-up (*n* = 435)
β-Blocker, *n* (%)	263 (44)	248 (53)	264 (44)	210 (49)
ACEi or ARB, *n* (%)	297 (50)	256 (54)	301 (50)	213 (49)
Loop diuretic, *n* (%)	136 (23)	118 (25)	125 (21)	29 (18,)
MRA, *n* (%)	97 (16)	25 (5)	99 (16)	11 (2.5)
Statin, *n* (%)	308 (51)	239 (51)	300 (50)	205 (47)
Anti-platelet, *n* (%)	199 (33)	127 (27)	202 (34)	125 (29)
Anticoagulation, *n* (%)	210 (35)	185 (43)	203 (34)	148 (34)
Digoxin, *n* (%)	48 (8)	37 (8)	38 (6)	30 (7)
CRT upgrade, *n* (%)	0 (0)	7 (1.2)	0 ()	4 (0.6)

ARB, angiotensin II receptor blocker.

One participant randomized to echocardiographic screening had no diagnostic images obtainable but was nevertheless included in their allocated group. In the 600 people allocated to echocardiography screening, the mean LVEF was 50% (10%), with 201 (34%) individuals identified as having LVEF < 50%. The prevalence of LVEF < 50% was similar across sites such that 101 (34% of those screened) were seen in the combined HF and devices clinic at site 1 and 100 (33% of those screened) received primary-care-led management (sites 2 and 3) (Fig. 1).

### Primary outcome measure

Participants were followed for a median of 31 months (interquartile range (IQR) = 30, 40 months) with a minimum follow-up for all patients of 12 months. Univariate and multivariable predictors of the primary outcome are demonstrated in Extended Data Tables 1 and 2 and included age (odds ratio (OR) = 1.07, 95% confidence interval (CI) = 1.05, 1.09), overt ischemic heart disease (IHD; OR = 1.52, 95% CI = 1.15, 2.02) and log(N-terminal pro-brain natriuretic peptide (NT-proBNP)) (OR = 1.69, 95% CI = 1.46, 1.96).

**Fig. 2:**
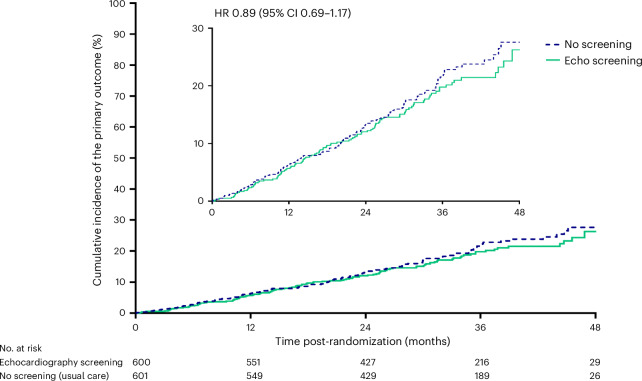
Time free of all-cause mortality or HFH by randomization group. Kaplan–Meier curve demonstrating the primary outcome in those allocated screening by echocardiography (intervention) or no screening (standard care).

The primary outcome occurred in 106 of 600 (18%) people randomized to echocardiographic screening and 115 of 601 (19%) in the usual care group (hazard ratio (HR) = 0.89; 95% CI = 0.69, 1.17) (Fig. 2). The estimated treatment effect adjusted by statistically significant predictors of outcome (Extended Data Table 1) did not alter the results (HR_adjusted_ = 0.95; 95% CI = 0.72, 1.24).

### Secondary outcome measures

Out of 903 participants with complete 12-month medical therapy data (468 echocardiographic screening, 435 usual care), participants screened with echocardiography received more medical therapy optimization (118 diuretic, 247 β-blocker, 255 angiotensin-converting enzyme inhibitor (ACEi) initiation/titration episodes) than participants in the usual care group (79 diuretic, 211 β-blocker, 214 ACEi initiation/titration episodes). Device system upgrades to cardiac resynchronization therapy (CRT) were performed in seven (1.2%) patients in the echocardiography group and four (0.6%) of those allocated to usual care.

Of 1,201 participants, 1,198 completed quality-of-life questionnaires at baseline (pre-randomization) with 878 (73%) also completing questionnaires at the 12-month follow-up. The EuroQoL-5D (EQ-5D) scores were similar at baseline (*n* = 1,184, 99%) for transthoracic echocardiogram-guided care (*n* = 591) and usual care (*n* = 593) (0.77 ± 0.25 and 0.76 ± 0.24, respectively). Conditional on survival, the quality of life described by EQ-5D scores at follow-up (*n* = 855, 71%) did not change for either echocardiography screening (*n* = 444) or usual care (*n* = 411) groups (0.76 ± 0.24 and 0.73 ± 0.31). Analysis of covariance (ANCOVA), allowing adjustment for baseline EQ-5D visual analog score (VAS), showed that the mean difference in VAS of those receiving echo (*n* = 436) to those receiving no echo (*n* = 406) was 0.85 (95% CI = −1.38, 3.08).

### Exploratory outcomes

**Fig. 3:**
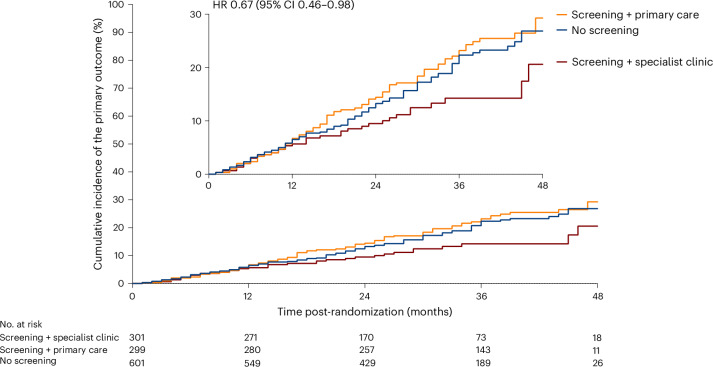
Time free of all-cause mortality or HFH by the clinical management pathway. Kaplan–Meier curve demonstrating the exploratory analysis of the primary outcome in those allocated screening by echocardiography (intervention) divided by management pathway (primary-care-led management or specialist clinic-led management) or no screening (standard care).

The prespecified exploratory analysis of the echocardiography screening group, according to the follow-up pathway, suggested that the groups, although nonrandomized for this comparison, were well balanced (Extended Data Table 3). Patients with LVEF < 50% were not randomly allocated to the follow-up care because this was a site-level pathway and only available at site 1. The rate of the primary outcome appeared lower in the site with access to the specialist clinic than in the two sites where the pathway of care included primary-care-led management (specialist clinic versus primary-care-led management: 12% versus 24%; HR = 0.67, 95% CI = 0.46, 0.98) (Fig. 3). This nonrandomized comparison is hypothesis generating and requires further validation. The difference between these two groups remained significant, even after adjustment for differences in the baseline variables, which, as shown in Extended Data Table 3, were atrial rhythm, systolic blood pressure (BP), resting heart rate, atrial fibrillation (AF) burden, ventricular pacing proportion and LVEF (HR = 0.62; 95% CI = 0.40, 0.97). On the other hand, there was no difference in outcome between those allocated to the usual care (no echocardiography) and those who received echocardiography but were not seen in the specialist clinic (HR = 1.01; 95% CI = 0.72, 1.40), including when the analysis was adjusted for baseline differences shown in Extended Data Table 3 (atrial rhythm, AF burden, base rate; HR = 0.88; 95% CI = 0.61, 1.27).

There were apparent differences in the medical management of those with impaired LV function at 12 months in participants randomized to echocardiographic screening and subsequently managed in a multidisciplinary HF and devices service compared with those receiving primary-care-based management. People with LVEF < 50% managed through the specialist service were almost 3× more likely at the 12-month follow-up to have undergone initiation or titration of β-antagonists (OR = 2.92, 95% CI = 1.43, 5.99) or a mineralocorticoid receptor antagonist (MRA; OR = 2.95, 95% CI = 1.01, 8.61) than people with LVEF < 50% in the echocardiography arm with primary-care-coordinated management. There were no differences in initiation or titration of ACEi therapy (OR = 1.64, 95% CI = 0.86, 3.14) (Extended Data Table 4).

### Sensitivity analysis

**Extended Data Fig. 2:**
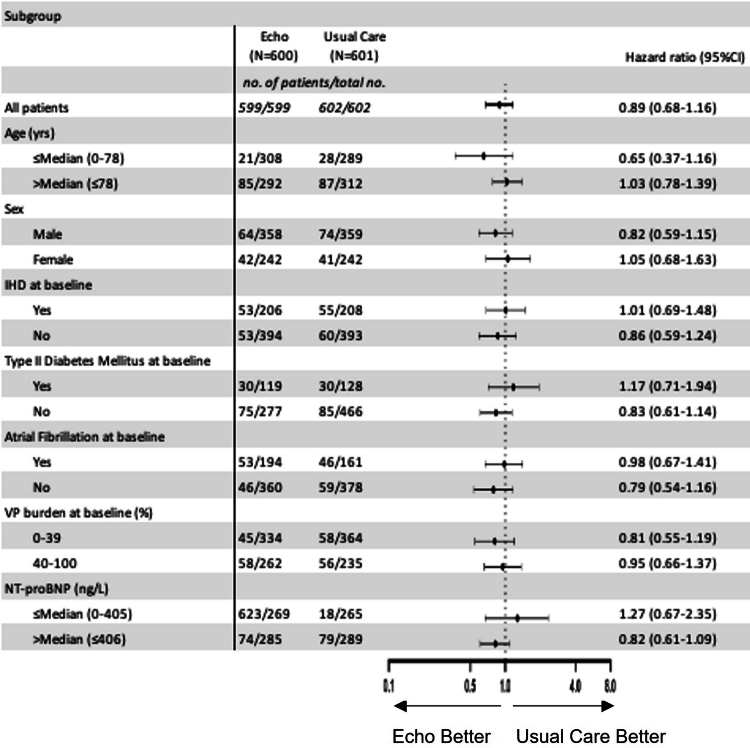
Time Free of All-Cause Mortality or Heart Failure Hospitalisation According to Prespecified Subgroup. Forest plot describing the primary outcome by relevant prespecified clinical subgroups showing no difference in primary outcome between those allocated screening by echocardiography and those allocated standard care.

Exploratory analysis of the primary endpoint within subgroups relevant to outcomes in this population (Extended Data Table 2), and associated with adverse outcomes in people with HF (diabetes mellitus and IHD)^12,13^, revealed that even in those at higher risk, the effect of echocardiography screening alone was neutral (Extended Data Fig. 2).

## Discussion

OPT-PACE tested whether a screening test, in this case an echocardiogram to identify LVSD, in people with a risk factor, in this case a pacemaker, is sufficient to improve outcomes. The present study was pragmatically delivered, collating additional information on two possible pathways of care of people with a positive result. As patient pathways for enhanced care differed at the site level, it was not possible to randomize patients between the specialist clinic and primary care.

OPT-PACE provides three important findings. First, in a contemporary population with pacemakers implanted for bradycardia but not yet known to have HF, routine echocardiographic screening identifies impaired LV function in around a third. Second, simply embedding echocardiography into a pacemaker service does not, in itself, lead to improved clinical outcomes. Third, a nonrandomized, planned exploratory analysis suggests that a pathway of care that includes coordinated optimization of medical therapy for HF within a multidisciplinary clinic, screening for and treating pacemaker-associated LV dysfunction might improve clinical outcomes, although this analysis is hypothesis generating and the results require further validation.

Consistent with our data, previous studies have shown that people with pacemakers implanted for bradycardia have a risk of impaired LV function (LVEF < 50%) or overt HF far higher than the general population^14^. Those with impaired LV function have a lower quality of life, higher hospitalization rate and poorer prognosis than those with a pacemaker and normal LV function^7^. Observational studies in people with pacemakers have consistently shown that the degree of LV dysfunction is related to the RV pacing burden^7,15^ with a linear relationship to risk of HF events and cardiovascular death^16^ and that LV impairment is more frequent in patients with pacemakers who also have underlying IHD or myocardial fibrosis^7,17^.

Although the adverse effects of RV pacing on LV function, and the fact that many people with standard pacemakers have LV dysfunction, have been appreciated for many years^14,16^, the medical management of pacemaker-associated HF is under-investigated. Optimal care in patients with impaired LV function includes renin–angiotensin–aldosterone system inhibitors^18^ and β-adrenoceptor antagonists^19,20^, with proven favorable effects on LV remodeling^21^ and patient-oriented long-term outcomes^22^. Moreover, a program with a protocol of initiation and titration of medical therapy for people with HFrEF provided in a specialized setting is associated with better outcomes^23^. Most of the phase III trials of medical therapy for HFrEF did not actively exclude people with standard pacemakers, but, although around 9% of people with HFrEF have a standard pacemaker^24,25^, none of the trials reported analyses for outcomes in this subgroup. Hence, as a result of a lack of data, guidelines make no particular mention of the investigation and management of people with pacemakers at risk of, or with proven, HFrEF except to comment on the use of algorithms to limit RV pacing^26^, and the potential benefits of upgrading standard pacemakers to CRT in the presence of symptoms, LV dysfunction and a high requirement for ventricular pacing^11,27^.

OPT-PACE has confirmed that a coordinated screening program involving routine echocardiography will detect previously undiagnosed impaired LV function in around a third of people with pacemakers implanted for bradycardia. The present study was not designed to determine the etiology of the LV dysfunction or whether it was primarily the result of RV pacing, but rather to determine whether screening for LV dysfunction led to improved outcomes in patients with standard pacemakers. The primary combined outcome of time to first HFH or death did not differ between those randomized to the usual care and those randomized to echocardiographic screening.

A prespecified exploratory analysis was included to explore whether a care pathway, involving a specialized HF and devices clinic tasked with patient-centered care, education, and initiation and titration of medical therapy for patients found to have an LVEF < 50%, as proposed in HF guidelines and a recent position paper, could be of additional benefit^28–30^. The present data revealed that those randomized to the screening arm who received management via the specialized service had a significantly lower event rate than either of the other two groups. This outcome could be the result of a combination of education around HF and the improved provision of medical therapy for HF, especially titration of β-antagonists. Higher doses of neurohormonal blockade are associated with improved outcomes, especially in people with comorbidities^12,31^. The trial was not, however, designed to definitively answer this question, which will require further validation.

Hence, OPT-PACE demonstrates that simply embedding echocardiographic screening into routine pacemaker follow-up services is not sufficient to improve patient outcomes, possibly contributed to by a highly heterogeneous response to the echocardiography result. It is possible that, to achieve better outcomes, echocardiography should form an integral part of a coordinated pathway of care that includes access to specialist HF expertise, education and coordinated patient-tailored initiation and up-titration of medical therapy for people with a standard pacemaker found to have impaired LV function, as for patients who undergo CRT^28^, although this hypothesis remains unanswered.

OPT-PACE is noteworthy in that studies testing the effects of diagnostic and therapeutic interventions in a single trial are uncommon. One example in the field of HF of a randomized controlled trial that combines screening with treatment is the randomized controlled STOP-HF study, which tested the utility of screening for HF in an asymptomatic population using natriuretic peptides, followed by optimization of medical therapy in a specialized clinic, on clinical outcomes^32^. However, OPT-PACE differs markedly from previous trials in that it targets an at-risk patient population and then explores two pathways of care on patient-oriented outcomes for those with the condition, thereby offering information not only on the effects of screening itself but also exploratory information on the response to it.

Guidelines recommend natriuretic peptides as an appropriate method of screening for HF in people with symptoms of breathlessness or fatigue^29,30^. We have previously explored the use of BNPs to identify HF in a pacemaker population^33^, but found modest specificity for LVSD. Hence, despite data that echocardiographic screening of the asymptomatic general population has a low positive rate and does not lead to improvements in outcomes^34^, the higher rate of LV impairment in the pacemaker population and the adverse outcomes associated with this, along with the therapeutic information provided by echocardiography, underpinned our decision to use this as our screening test. Further analysis of the OPT-PACE dataset, combined with ongoing observational studies including our previous work^7^, might serve to identify a cohort at highest risk of prevalent LV dysfunction in whom a tailored echocardiographic screening program might be particularly beneficial.

There are several limitations to the present study. OPT-PACE recruited people from three hospitals within a single region in the United Kingdom. Although this may limit generalizability compared with international models of care, the baseline demographic data suggest that our study is representative of a population treated with pacemakers for bradycardia. We utilized digital data extraction for follow-up. Hospitalization data were not available through a single system at the time of the study, such that patient admissions to other hospitals may be incomplete. However, the distribution of hospital facilities across the United Kingdom means that care is usually delivered by a single organization in a particular locality, making it unlikely that participants would be hospitalized elsewhere. Moreover, we do not envisage any ascertainment bias for one or other group owing to this. UK national mortality data are updated daily across the entire country, so the mortality data are reliable. There is an appreciable delay to the clinical effects of medical therapy for LV dysfunction that is longer for people who are less symptomatic^35,36^. Hence, longer follow-up might have shown greater effects on patient-oriented outcomes. OPT-PACE was designed and largely completed before the routine use of sacubitril–valsartan and sodium glucose transport protein 2 inhibitors for HFrEF. It is reasonable to propose that the effects of the specialized clinic are likely to have been greater with these agents as additional standard therapy.

Although allocation to echocardiography was randomized, it was not possible to randomize allocation to the follow-up pathway within the present study, given that this was a site-level pathway available in only one site. Hence, the results of the prespecified exploratory analysis describing the effect of the specialist clinic are hypothesis generating and require further research to provide independent validation.

In conclusion, the present study has shown that a third of people with a pacemaker implanted for bradycardia have impaired LV function. Implementing a pathway of care that includes only echocardiographic screening does not improve clinical outcomes. Further research will be required to determine whether a specialist clinic combining device and medical management improves outcomes.

## Methods

### Trial design and ethical approval

The study design and protocol have been published previously. OPT-PACE was a multicenter, randomized, open-label, parallel group trial conducted in three hospitals in the United Kingdom^37^. The trial design set out to test the clinical effects of screening echocardiography for impaired LV function in a population at risk, using a randomized controlled methodology. A prespecified, nonrandomized exploratory analysis to explore the effects of two different pathways of care for those identified as having impaired LV function was included. All participants provided written, informed consent and the trial was conducted according to principles outlined in the Declaration of Helsinki, having received full ethical approval from the Health Research Authority (South Yorkshire Research Ethics Committee: no. 12/YH/0487).

### Trial participants

Participants were eligible to take part if they had a pacemaker implanted for bradycardia at least 12 months previously due to any indication, according to the clinical guidelines in place at the time^38^, and were attending routine follow-up at three centers in the United Kingdom. People were ineligible if they were known to have HFrEF, had implantable cardioverter defibrillator or cardiac resynchronization devices, were <18 years old, pregnant, already under the care of HF services or awaiting heart transplantation, or had an anticipated life expectancy of <1 year due to comorbidity or significant cognitive impairment.

### Trial procedures

All participants were approached by their clinical team at a routine appointment and all provided written, informed consent before any trial activities. At baseline (pre-randomization) each patient underwent a standard pacemaker interrogation, the medical history was recorded, blood was tested for full blood count, renal function and NT-proBNP, and quality of life was assessed using the EQ-5D questionnaire. Quality-of-life questionnaires during the follow-up period were sent by mail.

The trial design included two phases. First, after the completion of baseline procedures, participants were randomly allocated on a one-to-one basis to echocardiographic screening for LV dysfunction or the usual care (Fig. 1 and Extended Data Fig. 1), using a randomization schedule derived by an independent statistical service and accessed through a web-based system. Those allocated to echocardiographic screening underwent an assessment of LV function according to European Society of Cardiology criteria using Simpson’s biplane measures to determine the LVEF^39^.

### Intervention

Participants allocated the usual care and those in the echocardiographic screening arm with an LVEF ≥ 50% continued with standard pacemaker follow-up. For those found to have impaired LV function (LVEF < 50%), two pathways of care were applied in a nonrandomized fashion, according to center-level practice. In two of the three centers, the results of the echocardiogram were forwarded to the patient’s primary care physician. Subsequent treatment, including onward referral, was at their discretion, accepting that this approach would include considerable heterogeneity. In the third center, patients with an LVEF < 50% were referred directly to a specialized multidisciplinary clinic combining HF and devices therapy, where medical therapy optimization was led by a team of HF nurse specialists and cardiac physiologists in a coordinated program of patient-centered care, education and titration visits, as outlined in HF guidelines and a recent position paper^28–30^. In all three centers, the usual care included programming to avoid unnecessary RV pacing where appropriate, as previously published^40^.

### Outcomes

Long-term survival, hospitalization and medical therapy were assessed using hospital and primary care digital patient records, including National Health Service (NHS) national and local systems.

The primary outcome was a composite of the time to first HFH or death comparing those randomized to echocardiographic screening or the usual care. A prespecified analysis of the primary outcome was included to enable an exploratory assessment of the effects of a care pathway that included screening for impaired LV function and medical optimization delivered through a multidisciplinary specialized HF and devices service or the patient’s primary care physician. Secondary outcome measures were the provision of guideline-directed medical therapy for HFrEF and quality of life, measured at 12 months.

### Statistical analysis

OPT-PACE was powered to detect an absolute reduction in the primary outcome events from 15% in the usual care group to 9% in the echocardiography screening group. A primary event rate of 15% was anticipated in people randomized to usual care^12,22,37,41,42^ and it was assumed that a third of people in both arms would have LVEF < 50% (ref. ^7^). Based on contemporary data of combined medical therapy in people with impaired LV function^12,22^, a larger reduction in clinical events, from 15% to 7.5%, was assumed in people with LVEF < 50%, which would be diluted by people with LVEF > 50%. To detect a reduction in events from 15% to 9% (equivalent to an HR of 0.58, based on the effects of combination therapy for HFrEF^12,21,31,43^) using log(rank analysis) with an overall type 1 error rate of 0.05 (two-sided analysis) and a power of 0.90, a total of 146 events were required to be observed in at least 1,070 participants (nQuery Advisor v.3.0, assuming 18-month recruitment and 12-month follow-up). The target recruitment was increased to 1,200 participants in anticipation of a drop-out rate of 10%.

Time to first HFH or death was calculated from the date of randomization to the date of the first event or the date of censor set at October 2017, when all participants had had a minimum of 12 months of follow-up. Event-free survival estimates were calculated using the Kaplan–Meier method compared across randomized groups using log(rank testing) in an intention-to-treat analysis.

Exploratory descriptive analysis in those allocated echocardiography, specified a priori in the statistical analysis plan, reported outcomes in the group with access to the specialist clinic and those treated by their primary care physician.

Multivariable analysis of the primary outcome assessed the influence of patient baseline characteristics using Cox’s proportional hazards regression modeling. Variables considered for selection were those previously reported in this population^7^ and included age, previous history of overt coronary artery disease, atrial rhythm, log(NT-proBNP), ventricular pacing burden, sex and the presence of diabetes mellitus. Adjusted treatment effects are reported.

Primary data storage was on Microsoft Excel (v.15) and primary analyses were performed using SAS. Exploratory analyses used SPSS statistical software. The attainment of medical therapy was assessed using Pearson’s *χ*^2^ analysis. Quality-of-life data between the randomized groups were reported descriptively and analyzed using ANCOVA. The statistical analysis plan is available as a Supplementary Note.

### Reporting summary

Further information on research design is available in the Nature Portfolio Reporting Summary linked to this article.

## Online content

Any methods, additional references, Nature Portfolio reporting summaries, source data, extended data, supplementary information, acknowledgements, peer review information; details of author contributions and competing interests; and statements of data and code availability are available at 10.1038/s41591-024-03265-3.

## Supplementary information

Supplementary Information Statistical analysis plan.

Reporting Summary

## Figures and Tables

**Extended Data Table 1:**
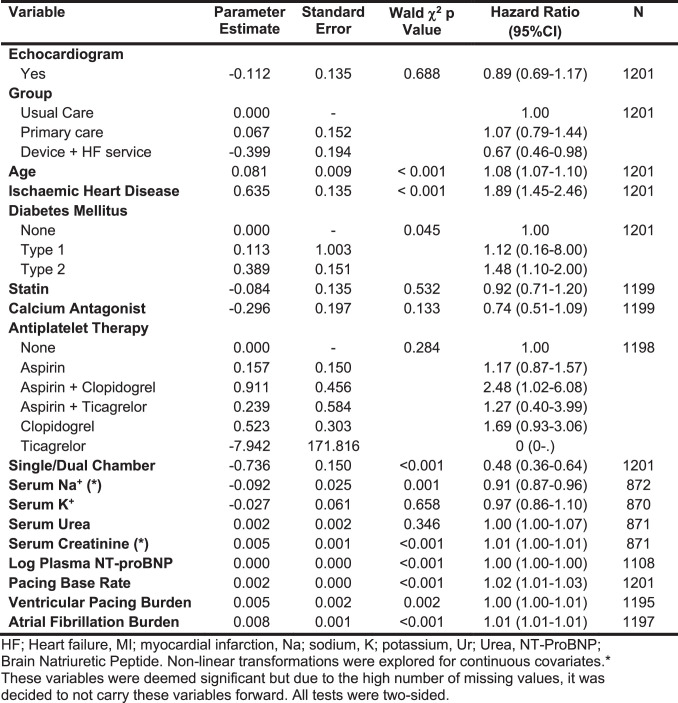
Univariate estimates to event-free survival

**Extended Data Table 2:**
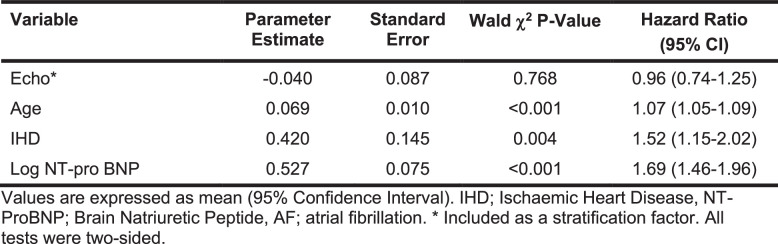
Multivariable analysis (Cox’s regression, adjusted for randomization to echocardiography) for time to survival from HFH or death

**Extended Data Table 3:**
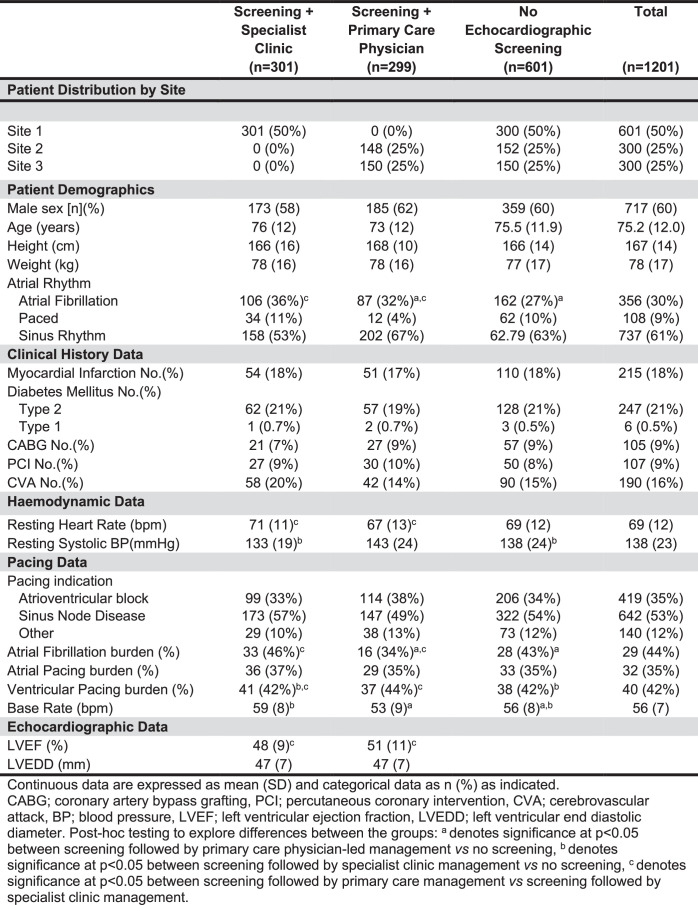
Patient demographics at baseline by care pathway

**Extended Data Table 4:**
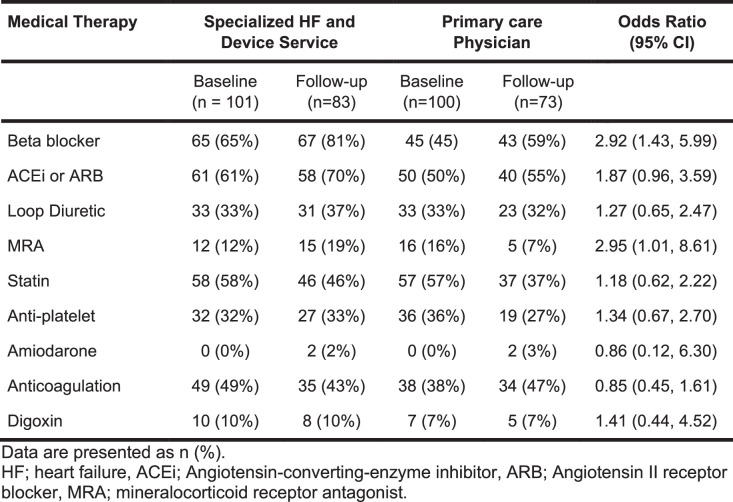
Drug therapy of patients with LVSD at baseline and after a 12-month follow-up by care pathway

## Data Availability

Individual participant data that underlie the results reported in this article will be available after de-identification (text, tables, figures and appendices) beginning 9 months and ending 36 months after article publication. Investigators requesting access will require a methodologically sound proposal approved by an independent review committee identified for this purpose to achieve the aims in their approved proposal. Data will be provided within 3 months of a request. Proposals should be directed to the corresponding author (K.K.W.).
