# Supplementary material for: Echocardiographic screening for heart failure and optimization of the care pathway for individuals with pacemakers: a randomized controlled trial
Source: Nat Med. 2024 Sep 19;30(11):3303–9. doi: 10.1038/s41591-024-03265-3 (PMC11564098; doi:10.1038/s41591-024-03265-3)
Supplement: Supplementary file 1 — Statistical analysis plan. [file 41591_2024_3265_MOESM1_ESM.pdf]

nature medicine

Supplementary information https://doi.org/10.1038/s41591-024-03265-3

Echocardiographic screening for heart failure and optimization of the care pathway for individuals with pacemakers: a randomized controlled trial

In the format provided by the authors and unedited

Clinical Trials Research Unit (CTRU) University of Leeds

Final
Statistical Analysis Plan

OPT-PACE

Version 1.0

February 2019

Trial Statistician:	Alasdair Fellows
Supervising Statistician:	Deborah Stocken
CTRU Scientific Lead:	Deborah Stocken
Data Manager/ Senior Data Manager:	Maria Paton
Chief Investigator:	Klaus Witte

Copyright © 2007 CTRU, University of Leeds. All rights reserved.

Table of Contents

Amendments **2**
1 Introduction **3**
1.1 Background 3
1.1.1 Pilot Study 3
1.2 Design 3
1.2.1 Blinding 3
1.3 Aims 3
1.4 Sample size and expected accrual 4
1.5 Planned analyses 4
2 Endpoints **4**
2.1 Primary Outcome Measure 4
2.2 Secondary Outcome Measure 4
2.3 Derivation of Outcome Measures 4
2.3.1 Primary Outcome Measure 4
2.3.2 Secondary Outcome Measure 5
2.4 Exploratory Analysis 5
2.5 Missing data 5
3 Populations **6**
3.1 Eligibility 6
3.1.1 Inclusion 6
3.1.2 Exclusion 6
3.2 Intention to treat population 6
3.3 As treated population 6
4 Data Handling **6**
4.1 Data validation 6
5 Data Analysis **7**
5.1 General calculations 7
5.2 Baseline Characteristics 7
5.3 Recruitment and Compliance 7
5.4 Analysis 8
5.4.1 Primary Analysis of the Primary outcome measure 8
5.4.2 Secondary Analysis 8
5.4.3 Exploratory analysis 9
6 Reporting and Dissemination of the Results **10**
7 References **10**
8 Appendices **10**
Appendix A Risk Model 10
Appendix B Baseline draft tables 11

Amendments

Copyright © 2007 CTRU, University of Leeds. All rights reserved.

1 Introduction

1.1 Background

This statistical analysis plan provides guidance for the final analysis of the OPT-PACE trial and is based off the most recent protocol paper for the study which is version 2.0, dated DEC 2018.

The OPT-PACE (OPTimising PACEmaker therapy) trial has been designed to address the issues that come from patients who have pacemaker implantations for bradycardia. It aims to fulfil two major goals. The first is to validate a HF risk stratification model based on simple variables available in pacemaker clinic with a secondary aim to will establish whether NT-proBNP improves this risk stratification model. Subsequently, we will assess the impact on quantify time to all-cause mortality or heart failure hospitalisation medical therapy, device programming and patient quality of life of identifying people with HF in pacemaker clinic. .

1.1.1 Pilot Study

The design of the OPTPACE trial is informed by an observational cohort [1] including 491 patients listed for a pacemaker generator replacement in a single tertiary centre (Leeds Teaching Hospitals NHS Trust) invited for pacing therapy information, diagnostic pacing data and an echocardiogram.

Multivariable analyses identified a number of simple clinical variables; high percentage RV pacing, high serum creatinine, and previous myocardial infarction as potential independent predictors of LV systolic dysfunction to identify patients who may benefit from a more comprehensive review. This risk model requires external validation planned within the OPT-PACE trial.

1.2 Design

OPT-PACE is a multicentre, non-blinded randomised controlled trial with two parallel arms funded by the National Institute for Health Research (NIHR) in the UK (NIHR-CS-2012-032) and registered with the Clinical Trials registry (NCT01819662).

The study is recruiting 1200 patients across 3 difference centres, one tertiary centre, Leeds Teaching Hospitals Trust, and two district centres; Harrogate District Foundation Trust and Bradford District Trust. With Leeds, recruiting 600 patients and Harrogate and Bradford simultaneously recruiting 300 patients each All patients will be followed up for a minimum of 12 months, with data being collected at baseline and then a 12 month follow-up appointment. Patients are simply randomised 1:1 either receive; the usual standard care or an echocardiographic guided care pathway, which is the noted intervention. Echocardiographic guided care pathway consists of enhanced (echocardiogram with primary care information) r optimized care (with referral to heart failure clinic) as pre-determined by each recruiting site.

1.2.1 Blinding

Due to the nature of the study and intervention, Practitioners and Participants in this trial cannot be blinded to allocation of the treatments. However, the outcome assessor will be blinded who has no prior knowledge of participant allocation. The outcome assessor will remain blinded throughout the course of the trial. Unbinding of the assessors will only be permitted in certain circumstances, such as serious AE’s.

1.3 Aims

To explore the clinical use of an echocardiogram to detect heart failure problems in patients who have an implanted pacemaker and to create models using this data to determine future risks for heart failure problems for patients in this population.

Copyright © 2007 CTRU, University of Leeds. All rights reserved.

The primary aims of the trial is to look at the time from randomisation to date of first event of all-cause mortality or heart failure hospitalization which is assessed by the Endpoint Review Committee.

The secondary aims of the study are:

- To validate a risk model to stratify people with a pacemaker for prevalent of future heart failure using simple clinical and pacing variables to predict the presence of left ventricular systolic dysfunction at baseline.
- The utility of BNP testing as a way to improve risk score for prevalent left ventricular systolic dysfunction and future admissions.
- Hospitalization rates and mortality across randomized arms.
- Utility of a diagnosis of cardiac dysfunction on achieving standard of care medications and doses.
- Quality of life as measured by the EuroQol (EQ5D) at baseline and 12 months post enrollment.

1.4 Sample size and expected accrual

The trial is designed to detect a reduction in hospitalization or mortality rate of 7.5% at one year in patients identified with cardiac dysfunction from 15% anticipated in patients randomized to the standard pathway [1]. Given approximately one third of patients in both randomised arms are estimated to have cardiac dysfunction, a 7.5% reduction would be diluted to a 9% overall reduction in the enhanced pathway arm. To detect a reduction in events from 15% to 9% (equivalent to a hazard ratio equal to 0.58) using log-rank analysis with an overall type 1 error rate of 0.05 (two-sided analysis) and a power of 0.90, requires a total of 146 events to be observed in at least 1070 participants (nQuery Advisor assuming 18 month recruitment) inflated to 1200 in anticipation of minimal drop-out.

1.5 Planned analyses

There is only a final analysis planned for this trial once all the data has been collected.

2 Endpoints

2.1 Primary Outcome Measure

- Time to mortality or first event of heart failure hospitalization.

2.2 Secondary Outcome Measure

- Validation of a risk model.
- Utility of BNP testing to improve risk scores.
- Hospitalization rates and mortality.
- Utility of a diagnosis of cardiac dysfunction on achieving standard care.
- Quality of life at baseline and 12 month follow-up point.

2.3 Derivation of Outcome Measures

2.3.1 Primary Outcome Measure

The primary outcome measure is the time to the first case of any cause mortality or admission for heart failure hospitalization. This is measured from the point of randomisation up until the time of event or 12 month follow-up point. If patients do not have an event they are censored at the time when they were last seen. The unit of measurement for this analysis will be in months.

Copyright © 2007 CTRU, University of Leeds. All rights reserved.

2.3.2 Secondary Outcome Measure

2.3.2.1 Hospitalization rates and mortality

This looks at the outcome measure of the hospitalization rate and mortality across the randomised arms of standard care and those who have an Echocardiography and enhansed care. The event for this measure is any case of mortality and any form of cardiovascular hospitalization. Patients will be censored at the point they were last seen. The rates will look at the groups over a long period of time and give different rates at different points.

2.3.2.2 Utility of a diagnosis of cardiac dysfunction on achieving standard care

The Utility of cardiac dysfunction will look at the drugs in which the patients received at baseline and compare them to the drugs they received at the 12 month follow-up point to see whether there was a change in medication and dosage. Will also look at achieving standard of care medications and doses by treatment group and whether those with Echo received better care due to the results of their scan, this will also look at whether the site and after care they received helps improve the care they receive.

2.3.2.3 Quality of life at baseline and 12 months follow-up point

The quality of life (QOL) outcome measure will look the patients scores from the EQ5D-3L and the QOL score. Comparing the values at baseline and at the 12 month follow-up point. This will show the changes in quality of life over time between the two appointments. It will look at the EQ VAS score, which is a score between 0 – 100, and also a calculated value for the 5 questions of; Mobility, Self-care, Activity, Pain and Anxiety/Depression, each of which are measured on 3 different levels. This score value is calculated by a basic algorithm [2].

2.4 Exploratory Analysis

2.4.1 Validation of a risk model

The risk model in which will be validated is a logistic regression model with three exploratory variables to predict the whether a patient is to have the event of LVEF < 50%. The risk models parameter estimates have been created in a previous study [1] and the OPT-PACE data will be used to validate the model, which is displayed in Appendix A.

2.4.2 Utility of BNP testing to improve risk scores

The addition of BNP testing will look at adding the variable BNP into the risk model to see if this improves the fit of the model. This variable will be looked at in a single score basis from a measurement of pg/ml and also look at it in a categorical form of putting patients into different BNP categories based on their BNP measurement.

2.5 Missing data

It will be reported those patients who did not attend the follow-up appointment and reason why they did not attend. For the analysis all data will be complete case and left as it is. For the outcome measures, time to event patients will be censored at point of last seen. Any other outcome measures, the data will be left as missing if it is not collected.

Copyright © 2007 CTRU, University of Leeds. All rights reserved.

3 Populations

3.1 Eligibility

3.1.1 Inclusion

- Patient can provide written consent.
- The patient has an implanted pacemaker for bradycardia.

3.1.2 Exclusion

- Under the age of 18 years old.
- The patient is Pregnant or may believe they are pregnant.
- The patient has an implanted cardioverter defibrillator.
- The patient has a Cardiac resynchronisation device.
- If they are waiting for a heart transplant.
- The patient has a severe co-morbidity with life expectancy of <1 year.
- The patient has a significant cognitive impairment and under the care of heart failure services.

3.2 Intention to treat population

An Intention-to-treat analysis (ITT) will be the primary method for analysing and summarising trial data. The ITT population is defined as all randomised participants, regardless of if they are ineligible, withdraw, do not comply with the protocol, are lost to follow-up or do not receive any study treatment. Only participants who withdraw consent for their data to be used in the trial, or for whom written informed consent for the full trial has not been received, will not be included in the ITT population. The ITT population will be analysed and summarised according to the treatment they were randomised to receive.

3.3 As treated population

The as treated population of the OPT-PACE study is all patients being reported with the treatment in which the received. This will act as the safety population, which will give all patients who have any adverse events or reactions.

4 Data Handling

4.1 Data validation

The data is entered through those who perform baseline and follow-up assessments on to an excel spreadsheet. This data is then read and formatted in SAS by the trial statistician. The raw data contains little to no formatting on it so in SAS a programme will need to be written to format all the data to ensure it can be used in the correct analysis. This will be changing all numeric variables into the correct format and ensuring any data variables are inputted as the correct 'ddmmyyyy' format in SAS to allow them to be used in analyses. An issue in which have arisen is missing dates are stored as either the date 31/12/2020 or the number zero which causes a problem in SAS as the value of zero is the baseline date value of 1/1/1960. So before the data is formatted these values will need to be noted and converted to missing data and formatted correctly. We will also look at to ensure any categorical variables are formatted the same way which includes checking spelling and all follow the same format.

Once the data has been formatted and cleaned correctly, validation checks will be done to ensure that the data is as it should be and all makes sense. Anything found to be out of place will be reported back to those who collected the data to try resolve the issues. Then any corrections which can be made to the dataset will

Copyright © 2007 CTRU, University of Leeds. All rights reserved.

be done and sent back to perform further validation checks.

Validation check which will be performed on the data set are:

- Checking eligibility of all randomised patients.
- Checking for a balance in centres to ensure 1:1 recruitment rate was met and also that right amount of patients were recruited in each centre.
- Checking for sequential dates with each patients data.
- Checks for missing data and if some variables have more missing values that others.
- Checking for any unusual and outlying data.

5 Data Analysis

The final analysis is responsibility of the Trial Statistician to perform.

5.1 General calculations

Unless otherwise stated, all percentages will be calculated using the total number of patients within the specified analysis population as the denominator (i.e. including all patients with missing data for that variable). All percentages, means, medians and ranges will be given to 1 decimal place. All statistical tests performed will be two sided tests at a 5% significance level and giving the 2-sided 95% confidence intervals. These tests and confidence intervals will be displayed to 2 decimal places, along with standard errors, standard deviations and parameter estimates. Any presented p-value will be given to 3 decimal places, any p-value lower than 0.001 will be shown as <0.001.

Summary statistics will show the number of non-missing items as well as the number of missing items. For categorical variables tables will be made to show the quantity of each variable. Non-quantifiable values would be reported as an inequality and limits of quantification value would also be reported. In each analysis relevant population summary statistics will be presented by treatment arms and also for the whole analysis population, unless explicitly stated otherwise.

All analyses will be done in SAS unless otherwise stated.

5.2 Baseline Characteristics

A table of baseline characteristics will be displayed before any endpoint analysis begins to look at the balance in groups between the randomised arms, since no real stratification methods were used at randomisation. These will compare a range of baseline characteristics in each arm and also look at difference between each centre. No actual statistical tests will be performed here though, these will all be simply observant checks between the randomised arms. A draft of these tables are displayed in Appendix B.

5.3 Recruitment and Compliance

A CONSORT diagram will be used to show the flow of patients throughout the study from randomisation to analysis. This will show patients who are randomised into what arm, and those who may withdraw from the trail. Summaries of the time of recruitment and the time of the trial will be given.

Summaries of the compliance of the patients and the treatment in which they all received will be shown in tables for each randomised arm. If any reasons why patients were not compliant or why they received a different treatment are collected then they will also be summarized.

Copyright © 2007 CTRU, University of Leeds. All rights reserved.

5.4 Analysis

5.4.1 Primary Analysis of the Primary outcome measure

Summary of the number of patients who had the primary outcome measure event will be presented, split between treatment arms. The details of the primary outcome measure are listed in section 2.3.1. The cause of event will also be summarised to show whether patients occurred heart failure hospitalization or mortality and the cause of mortality.

Summary statistics of the 12 month survival estimate along with relating Kaplan Meier curves for the two groups will be displayed. This will include number of patients who are censored. This analysis will use the intention to treat population. Then a log rank test will be performed on these curves to determine whether there is a difference between the two groups.

As an exploratory analysis, we will look at those patients on the enhanced care pathway and the site in which they went too. Due to Leeds providing a different enhanced care to those at Bradford and Harrogate. The Kaplan Meier curves will be looked at for Leeds, Bradford and Harrogate, separated for the treatments in which the patients received.

To determine if there is any difference between the two treatment arms, a univariate Cox Proportional Hazards model will be made to calculate the hazard ratio for the treatment and the 95% Confidence interval. This model will use the events of the 12-month primary outcome measure and survival times. This will give us the hazard of having the event for each treatment arm at the 12 month follow-up period. The Cox model assumptions will be checked by looking at; Proportional hazards assumption by looking at the log cumulative hazard plot for the covariates, linear covariate relationships and independence of observations. If they are not met, an accelerated failure time model will be looked at being made.

A further exploratory analysis will create a multivariable model using the treatment effect and other baseline characteristic variables such as age, gender, site, co-morbidities, type of device, NYHA class, medication, blood measures and underlying rhythm to see what effect they have on the primary outcome measure of heart failure hospitalization and any case of mortality at 12 months. These variables will be explored for their interactions with the treatment effect. Any continuous variables will be looked at using fractional polynomials to improve their fit into the model.

To select which other baseline characteristic variables to include in the model alongside treatment, the full list of baseline variables will be made into a set of simple hazards model with the variable as the only exploratory variable, the p-value will be looked at to determine whether it is less than 0.15. If it is it will included into a larger model. Forwards variable selection will be used to then build up a model using the values of AIC as a decision whether to include a variable or not.

5.4.2 Secondary Analysis

5.4.2.1 Hospitalization rates and mortality across randomized arms

This secondary outcome measure will use the Intention to treat population.

Summary statistics will be reported for the secondary outcome measures of hospitalization and mortality rates. These will give the rates for both the randomisation arm. They will look at the rates at 12 month and other further time points at each year. This will look at the rates of heart failure hospitalization and also cardiovascular hospitalization. Kaplan Meier curves will be displayed for each randomised arm for the rates of hospitalization and mortality for the full length of the trial, with displays giving the values at the 12 month period.

Copyright © 2007 CTRU, University of Leeds. All rights reserved.

Comparing the rates of cardiovascular hospitalization and all-cause mortality between the two arms will be done by using simple rate ratios and there confidence intervals. Looking at how many events happen for each patient from point of randomisation to the 12 month follow-up period.

Further exploratory analysis will look at the effect of other baseline factors to the rate ratio for patients. Forest rate ratio plots for each baseline factor and their confidence intervals will be made to display the effects on rate in which they have and the difference in treatment arms and difference in rate from between other variables.

5.4.2.2 Utility of a diagnosis of cardiac dysfunction on achieving standard of care

Summaries of the drugs in which patients received at baseline and at the 12 month follow-up point will be reported and given by the treatments in which the patients were on and the site they attended. It will also show summary statistics of the drugs dosages given for each treatment and site, before and after. The number of patients who changed or remained on the same drugs over the 12 months will be looked at and if so the change in dosages which they had.

Simple unadjusted odds ratios will be made to see whether those on the enhanced care pathway are more likely to be receiving drugs at 12 months then those not on the standard pathway.

A set of linear models will be used to look at whether the dosages which patients received at 12 months differs to what they received at baseline for each drug and that if the dosages of the drugs is also similar between the two groups at 12 months. These model will adjust for what the patient baseline dosage value is, the treatment they received and the site in which the patient attended. Each model will only use patient which were receiving the drug at the 12 month follow up point. Each model will have these assumptions checked; Normality of response variable, No homoscedasticity and residuals are normally correlated.

5.4.2.3 Quality of life as measured by EQ5D at baseline and 12 months

Summaries of both EQ5D Quality of life scores at baseline and 12 months will be presented. These summaries will include the overall QOL utility and the score formed from the five categories in the EQ5D-3L specified in section 2.3.2.3. These will be reported by treatment arms using the intention to treat population.

A plot showing patients change over time will be made with a plot of their total QOL score at baseline compared to the score given at the 12 month follow-up point. The same graph will be plotted for the EQ5D-3L scores, with a change over time. There will be different plots for both treatments with each treatments mean regression line plotted onto the graph.

A linear model will be fitted for the QOL score and EQ5D-3L Score at 12 months. These models will look at the change from baseline values of QOL score and EQ5D-3L score and adjust for the treatment in which each patient received. These models will be adjusted further with the addition of baseline exploratory variables; age, gender and site. For this model assumptions will need to be checked to ensure the residuals are normal, the residuals are independent and no evidence of homoscedasticity.

5.4.3 Exploratory analysis

5.4.2.1 Validating the risk model

Summaries of the patients who received and echo will be given and the statistics of their values of LVEF, and number of patients which have the event of LVSD (LVEF <50%). Summary statistics of the exploratory variable values will be given and the number of complete cases for the model will be noted as only a

Copyright © 2007 CTRU, University of Leeds. All rights reserved.

complete case will be used in the model.

Then using the given model in Appendix A, the data from OPT-PACE will be fitted to it to give risk scores for all patients. This model used a risk cut off point of 20% to determine whether a patient had the event or not. With the scores the amount of events that the model predicted will be given. Then the goodness of fit will be given by comparing the predicted events by the actual observed events. Hosmer-Lemeshow method will be used to determine how well the observed values match with the expected values. ROC Curves will also be displayed to give the C-statistic of the model.

Once we have determined that this model is validated, a new model will be created using the OPT-PACE data to look at the treatment effects of the model and to see if they are similar or differ to those in the pilot model. Assuming that the parameter estimates will be similar in the newly created model to the old model, this model will be used later in the analysis to allow the addition on the BNP variable. To validate this model we will use cross validation and use the same validation methods used on the pilot data.

5.4.2.2 The Utility of BNP testing

Summary statistics of the BNP Variable will be looked at. Its relationship of the BNP score and the other variables in the pilot model will be assessed.

The variable BNP will be added into the newly created risk model. Then the new parameter estimates, confidence intervals and p-values will be displayed to ensure that all model variables are significant. BNP will be looked at as a first order factional polynomial to see whether it would improve the fit of the continuous variable in the risk model.

This model will be validated using the same methods used to validate the new model in section 5.4.2.1.

6 Reporting and Dissemination of the Results

A Statistical report will be created. The results will aimed to be published in a cardiovascular journal and presented at cardiovascular conferences.

7 References

1. John Gierula, R.M.C., Haqeel A Jamil, Rowenna J Byrom, Zac L Waldron, Sue Pavitt, Mark T Kearney, Klaus KA Witte, *Patients with long term permanent pacemakers have a high prevalence of left ventricular dysfunction.* Journal of Cardiovascular Medicine, 2014. **16**(11): p. 743-750.
2. Dolan, P., *Modeling Valuations for EuroQol Health States.* Medical Care, 1997. **35**(11): p. 1095-1108.

8 Appendices

Appendix A Risk Model

				95% Confidence Intervals		
**Variable**	**Coefficient**	**Standard Error**	**Odds Ratio**	**Low**	**High**	**P Value**
Constant	-3.928	0.844				<0.001
MI (Yes)	1.297	0.490	3.66	1.41	9.57	0.008
%VP (Per %)	0.026	0.005	1.03	1.02	1.04	<0.001
Creatinine (per µmol)	0.016	0.006	1.02	1.00	1.03	0.011

Copyright © 2007 CTRU, University of Leeds. All rights reserved.

Appendix B Baseline draft tables

Variable		Echo	No Echo	All
N				
Site	Bradford			
	Harrogate			
	Leeds			
Age mean (SD)				
Height				
Weight				
Resting HR				
Resting Systolic HR				
Resting Diastolic HR				

Variable		Leeds	Bradford	Harrogate	All
Patient Echo	Echo				
	No Echo				
Age					
Height					
Weight					

Variable		Echo	No Echo	All
Generator Years				
Years since first Implant				
Symptom	A1. Unspecified			
	A2. Unknown			
	A3. Other			
	B1. Syncope			
	B2. Dizzy Spells			
	B3. Bradycardia			
	B4. Cardiac arrest			
	C1. Tachycardia			
	C1B. Palpitations			
	D1. None			
	D2. Heart Failure			
	D4. Chest Pain			
	Missing			
Pacemaker Technology	Dual			
	Single			
	Missing			
Atrial Lead	Passive			
	Active			
	Missing			
Ventricular Lead	Passive			
	Active			
	Missing			

Copyright © 2007 CTRU, University of Leeds. All rights reserved.

Approval of Analysis Plan

Clinical Trials Research Unit (CTRU)

The following Final analysis plan, February 2019 for the OPT-PACE study has been approved by the following personnel. Any signed amendments to the plan will be filed with this document.

Trial Statistician: Alasdair Fellows

Signature: ____

Date: ____

Supervising Statistician: Deborah Stocken

Signature: ____

Date: ____

Chief Investigator: Klaus Witte

Signature: 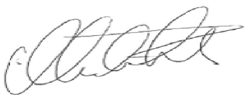

Date: 19th February 2019

Additional information:

Copyright © 2007 CTRU, University of Leeds. All rights reserved.
