# Supplementary material for: Echocardiographic screening for heart failure and optimization of the care pathway for individuals with pacemakers: a randomized controlled trial
Source: Nat Med. 2024 Sep 19;30(11):3303–9. doi: 10.1038/s41591-024-03265-3 (PMC11564098; doi:10.1038/s41591-024-03265-3)
Supplement: Supplementary file 2 — Reporting Summary [file 41591_2024_3265_MOESM2_ESM.pdf]

Corresponding author(s): Klaus K Witte

Last updated by author(s): Aug 22, 2024

Reporting Summary

Nature Portfolio wishes to improve the reproducibility of the work that we publish. This form provides structure for consistency and transparency in reporting. For further information on Nature Portfolio policies, see our Editorial Policies and the Editorial Policy Checklist.

Statistics

For all statistical analyses, confirm that the following items are present in the figure legend, table legend, main text, or Methods section.

n/a	Confirmed	
☐	☒	The exact sample size (*n*) for each experimental group/condition, given as a discrete number and unit of measurement
☐	☒	A statement on whether measurements were taken from distinct samples or whether the same sample was measured repeatedly
☐	☒	The statistical test(s) used AND whether they are one- or two-sided<br>*Only common tests should be described solely by name; describe more complex techniques in the Methods section.*
☐	☒	A description of all covariates tested
☐	☒	A description of any assumptions or corrections, such as tests of normality and adjustment for multiple comparisons
☐	☒	A full description of the statistical parameters including central tendency (e.g. means) or other basic estimates (e.g. regression coefficient) AND variation (e.g. standard deviation) or associated estimates of uncertainty (e.g. confidence intervals)
☐	☒	For null hypothesis testing, the test statistic (e.g. *F*, *t*, *r*) with confidence intervals, effect sizes, degrees of freedom and *P* value noted<br>*Give P values as exact values whenever suitable.*
☒	☐	For Bayesian analysis, information on the choice of priors and Markov chain Monte Carlo settings
☐	☒	For hierarchical and complex designs, identification of the appropriate level for tests and full reporting of outcomes
☐	☒	Estimates of effect sizes (e.g. Cohen's *d*, Pearson's *r*), indicating how they were calculated

Our web collection on statistics for biologists contains articles on many of the points above.

Software and code

Policy information about availability of computer code

Data collection	No specific software or code was utilised to collect the data. Data preparation was performed in SPSS version 28 (IBM, New York, USA)
Data analysis	Commercially available software, SPSS version 28 (IBM, New York, USA)

For manuscripts utilizing custom algorithms or software that are central to the research but not yet described in published literature, software must be made available to editors and reviewers. We strongly encourage code deposition in a community repository (e.g. GitHub). See the Nature Portfolio guidelines for submitting code & software for further information.

Data

Policy information about availability of data

All manuscripts must include a data availability statement. This statement should provide the following information, where applicable:

- Accession codes, unique identifiers, or web links for publicly available datasets
- A description of any restrictions on data availability
- For clinical datasets or third party data, please ensure that the statement adheres to our policy

Individual participant data that underline the results reported will be available after de-identification (text,tables, figures and appendices) beginning 9 months and ending 36 months following article publication. Investigators requesting access will require a methodologically sound proposal approved by an independent review committee, identified for this purpose to achieve the aims in their approved proposal. Proposals should be directed to the corresponding author

(K.K.Witte@Leeds.ac.uk) and any data that can be shared will be released with Sponsor approval via a data use agreement.
Responses will be aimed to be given within 3 months.

Research involving human participants, their data, or biological material

Policy information about studies with human participants or human data. See also policy information about sex, gender (identity/presentation), and sexual orientation and race, ethnicity and racism.

Reporting on sex and gender	Sex and gender distributions of the datasets have been reported and were collected based on self report.
Reporting on race, ethnicity, or other socially relevant groupings	Race was collected based on self-report. Analyses of primary or secondary outcomes by race or ethnicity were not planned a priori. Sex was included as a covariate in secondary analyses.
Population characteristics	Participants were recruited in 3 sites in the United Kingdom and were eligibile if they had a pacemaker implanted for bradycardia at least 12 months prior, had capacity to consent, and were over 18 years of age.
Recruitment	Consecutive, unselected potential participants were sent written information prior to their routine pacemaker follow-up and were approached for consent on the day of their appointment by the study team. Written informed consent was gained from all participants.
Ethics oversight	Ethical approval was given by the Health Research Authority (South Yorkshire Research Ethics Committee: 12/YH/0487)

Note that full information on the approval of the study protocol must also be provided in the manuscript.

Field-specific reporting

Please select the one below that is the best fit for your research. If you are not sure, read the appropriate sections before making your selection.

☒ Life sciences ☐ Behavioural & social sciences ☐ Ecological, evolutionary & environmental sciences

For a reference copy of the document with all sections, see nature.com/documents/nr-reporting-summary-flat.pdf

Life sciences study design

All studies must disclose on these points even when the disclosure is negative.

Sample size	A total of 1201 participants were included and randomised. The sample size was calculated to detect a reduction in clinical events from 15% to 9% based on previous evidence, using a log-rank analysis with an overall type 1 error rate of 0.05 (two-sided analysis), and a power of 0.90. A total of 146 events were required to be observed in at least 1070 participants assuming an 18month recruitment period (nQuery Advisor V3.0). Recruitment was increased to allow for a 20% drop-out rate.
Data exclusions	Exclusions from analysis were based on data completeness, numbers included are presented per analysis.
Replication	Not applicable as this is the first study to assess the utility of introducting echocardiography screening into a pacemaker follow-up service and to assess subsequent pathways of care.
Randomization	Participants were randomised in a 1:1 allocation to echocardiography or usual care. Subsequent management pathway was stratified by recruiting centre.
Blinding	No blinding was performed. This was an open label parallel randomised group trial.

Reporting for specific materials, systems and methods

We require information from authors about some types of materials, experimental systems and methods used in many studies. Here, indicate whether each material, system or method listed is relevant to your study. If you are not sure if a list item applies to your research, read the appropriate section before selecting a response.

Materials & experimental systems

n/a	Involved in the study
☒	☐ Antibodies
☒	☐ Eukaryotic cell lines
☒	☐ Palaeontology and archaeology
☒	☐ Animals and other organisms
☐	☒ Clinical data
☒	☐ Dual use research of concern
☒	☐ Plants

Methods

n/a	Involved in the study
☒	☐ ChIP-seq
☒	☐ Flow cytometry
☒	☐ MRI-based neuroimaging

Clinical data

Policy information about clinical studies

All manuscripts should comply with the ICMJE guidelines for publication of clinical research and a completed CONSORT checklist must be included with all submissions.

Clinical trial registration	ClinicalTrials.gov identifier NCT01819662
Study protocol	Full study protocol is available via Nature Medicine.
Data collection	Data were collected on-site by clinical research associates. Patients were recruited from June 2013 to November 2016 in 3 centres in the UK. Follow-up data were censored at on October 31st 2017. Data were collected from 1 tertiary and 2 district hospitals in paper format and entered onto a single digital database.
Outcomes	The primary outcome was time to first heart failure hospitalisation or all-cause death compared between intervention arms. Prespecified subanalysis included assessment of the effects of the subsequent care pathway. Secondary outcomes included the provision of guideline-directed medical therapy and quality of life.
